# An annotated selection of World Wide Web sites relevant to the topics in *Microbial Biotechnology*

**DOI:** 10.1111/1751-7915.12034

**Published:** 2013-02-15

**Authors:** Lawrence P Wackett

**Affiliations:** Department of Biochemistry Molecular Biology & Biophysics, BioTechnology Institute, University of MinnesotaSt. Paul, MN, 55108, USA

## Applied metagenomics of the watershed microbiome

http://www.genomebc.ca/portfolio/projects/environment-projects/applied-metagenomics-of-the-watershed-microbiome/

This page describes a project to use metagenomics for monitoring ecosystem and environmental health with respect to watersheds.

## Integrated microbial genomes: Microbiomes

http://img.jgi.doe.gov/cgi-bin/m/main.cgi

This database contains metagenome data sets and annotations allowing various applications of the data.

## Applied metagenomics to obtain novel cellulases

http://pubs.rsc.org/en/Content/ArticleLanding/2009/GC/b820157a

This paper describes a study to identify novel cellulases that are active in ionic liquids.

## Metagenomics of hydrocarbon bioremediation

http://www.plosone.org/article/info%3Adoi%2F10.1371%2Fjournal.pone.0030058

This article describes an oil spill in the Canadian arctic and a metagenomic study coincident with the ongoing hydrocarbon bioremediation project.

## Metagenomics following Deepwater Horizon oil spill

http://precedings.nature.com/documents/5733/version/1

This article deals with the recovery of microbial populations in two sites in the Gulf of Mexico prior to and after impact from the Deepwater Horizon oil spill.

## Metabolic reconstruction from metagenomes

http://bioinformatics.knowledgeblog.org/2011/06/21/a-survey-of-metabolic-reconstruction-tools-for-metagenomic-datasets/

Metabolic reconstruction is important in the use of genomic and metagenomic data. This blog to discusses differences in annotation tools and offers information on new tools.

## Applied metagenomics; seminar slides

http://blog.tshw.de/wp-content/uploads/2009/03/Applied-Metagenomics-Presentation.pdf

This set of PowerPoint slides gives some examples of application of metagenomic studies of microbes.

## Metagenomics for biofuels

http://www.biotechnologyforbiofuels.com/content/pdf/1754-6834-4-23.pdf

This study describes mining of metagenomic data for novel glycohydrolases that might be useful to deconstruct plant biomass and produce biofuels.

## Metagenomics for monitoring industrial processes

http://www.ecolyse.com/microbeid.php

This company offers services using metagenomics to monitor microbial populations with respect to biofouling, biocide effectiveness and hydrogen sulfide production; support services for drilling industries.

## Hydrocarbon metagenomics

http://www.hydrocarbonmetagenomics.com

Potential applications of the hydrocarbon metagenomics project are in oil sands remediation and stimulating coal bed methane generation.

## Microbiology in oil fields

http://www.spe.org/jpt/print/archives/2011/11/11Microbes.pdf

This article highlights microbiology relevant to the oil industry and includes such methods as metagenomic analysis.

## Time for metagenomic basis of therapeutics?

http://www.sciencemag.org/content/336/6086/1253

Microbes in the gut influence the efficacy or activation of oral drugs. This page links to an article discussing how metagenomics could help in this assessment.

## MetaPath

http://metapath.cbcb.umd.edu

This project was initiated to analyse metabolic pathways reconstructed from metagenomic data.

## Metagenomic view of xenobiotic metabolism

http://www.sciencedirect.com/science/article/pii/S1043661812001545

This article reviews literature on our current understanding of how complex microbial consortia degrade xenobiotic compounds in soil, water and the human intestine and the application of metagenomics to aid in these studies.

